# Sperm Inspection for In Vitro Fertilization via Self-Assembled Microdroplet Formation and Quantitative Phase Microscopy

**DOI:** 10.3390/cells10123317

**Published:** 2021-11-26

**Authors:** Yuval Atzitz, Matan Dudaie, Itay Barnea, Natan T. Shaked

**Affiliations:** Department of Biomedical, Engineering Tel Aviv University, Tel Aviv 6997801, Israel; yuvalatzitz@mail.tau.ac.il (Y.A.); matan.dudaie@gmail.com (M.D.); itay.barnea.1@gmail.com (I.B.)

**Keywords:** quantitative phase imaging, digital holographic microscopy, sperm cells

## Abstract

We present a new method for the selection of individual sperm cells using a microfluidic device that automatically traps each cell in a separate microdroplet that then individually self-assembles with other microdroplets, permitting the controlled measurement of the cells using quantitative phase microscopy. Following cell trapping and droplet formation, we utilize quantitative phase microscopy integrated with bright-field imaging for individual sperm morphology and motility inspection. We then perform individual sperm selection using a single-cell micromanipulator, which is enhanced by the microdroplet-trapping procedure described above. This method can improve sperm selection for intracytoplasmic sperm injection, a common type of in vitro fertilization procedure.

## 1. Introduction

Infertility is defined as the inability to achieve a successful pregnancy for at least 12 months [1]. As reported by the World Health Organization (WHO), nearly half of the cases of infertility are related to male infertility, and at least 30% of these cases are caused solely by malefactors [2,3,4]. To check infertility in males, semen quality is examined with different parameters such as sperm count, motility, and morphology of the cells [4,5]. Assisted reproduction technologies (ART), such as in-vitro fertilization (IVF) [6] and intracytoplasmic sperm injection (ICSI) [7], help increase the chances of successful fertilization. The isolation of sperm cells with normal morphological and motility characteristics can increase the chances of healthy pregnancies [8].

In conventional IVF, the oocytes are fertilized in a dish by motile sperm cells [9]. In contrast, ICSI involves selection of a single sperm cell from the sample using a cell micromanipulator, followed by injection of the selected cell into the oocyte [8,10]. Previous studies showed that sperm cells with high motility and normal morphology have higher fertilization potential [5,10].

Cell staining is not allowed in human ART. Without staining, sperm cells, as many other cells in vitro, are mostly transparent, and thus do not provide enough imaging contrast for internal morphological analysis. Embryologists consider and select sperm cells under bright-field microscopy based on their motility and external morphology. This is done in a non-quantitative manner while the sperm cells rapidly swim in a dish. In order to avoid losing a sperm cell due to its dynamic nature and before catching it for injection, the sperm characterization is completed by imaging it in a large field of view under low magnification (typically 10× or 20×), which then requires low-resolution imaging and further diminishes the ability of the clinician to well characterize the sperm morphological details. Overall, this process suffers from significant human errors, and the selection of the most potent sperm cells is still a challenge in the current practical procedures [3,8].

Qualitative phase microscopy techniques, such as differential interference contrast microscopy (DIC) and Zernike’s phase-contrast microscopy, create stain-free imaging contrast and detect details in the sperm cells that cannot be seen using bright-field microscopy [8,11,12,13]. However, these qualitative imaging techniques do not create substantial contrast on all the cell spatial points and suffer from imaging aberrations, such as shadows and halos, which might occlude important morphological details. Quantitative phase microscopy is a stain-free holographic-imaging method that uses interference between a beam passing through the sample and a mutually coherent reference beam to record the quantitative phase map of the cell. This map is proportional to the cell optical thickness or its optical path delay (OPD), which is induced from the cell refractive index values across its thickness at each point on the image [13,14,15]. Stain-free quantitative phase microscopy of sperm cells provides improved contrast that correlates well to that of stained sperm cells. Moreover, new parameters, such as the cell dry mass and volume, are now available for selection of potent cells [16,17,18]. Transport of intensity equation (TIE) microscopy [19] uses defocused images under white-light illumination and iterative calculations to retrieve the quantitative phase map, making it less attractive for real-time imaging of highly dynamic sperm cells. Alternatively, off-axis holography under coherent or partially coherent illumination can be used [16], providing a quantitative phase map from a single camera exposure without iterative calculations.

Microfluidic devices were previously used for single-cell analysis in general [20,21] as well as for the selection of sperm cells based on their motility in particular, typically disregarding the cell morphological analysis [22,23]. We previously integrated stain-free quantitative- phase microscopy with a disposable microfluidic device for the selection of individual sperm cells [8]. Using this method, sperm cells were sorted based on their morphology, as measured by quantitative phase microscopy, and then the most potent cells were directed to a dedicated reservoir using microfluidic pumps. After the sorting process, the clinician can choose a sperm cell from this reservoir and inject it into the egg. To ensure the separation of the cells while flowing inside the channel and avoid cell aggregation, the cells need space to spread out. Therefore, the concentration of the cells should be low, leading to low throughput of the analysis.

In this paper, we present a new method that provides high-magnification quantitative phase imaging of sperm cells that have been trapped in microdroplets so that the cells cannot escape after their analysis, followed by the extraction of the selected cells from the microdroplets using a cell micromanipulator. For this, we utilized a special microfluidic device that could trap cells individually in watery microdroplets separated with bio-compatible oil, where each microdroplet creates a microenvironment for individual cell analysis [24,25,26]. This is the first time, to the best of our knowledge, that such microdroplets have been used for motile sperm-cell trapping, imaged for analysis, and then followed by sperm extraction with a micromanipulator. This new technique has potential for clinical application, providing both quantitative imaging capabilities during ICSI and avoiding a situation where the clinician has to chase after potent sperm cells following their analysis.

## 2. Materials and Methods

### 2.1. Sperm Sample Preparation

Human samples were obtained after receiving Tel Aviv University’s institutional review board (IRB) approval, and signed consent forms from the sperm donors were obtained. The raw semen was incubated at room temperature for 1 h. Then, the sperm cells were isolated using the PureCeption Bi-layer kit (ORIGIO, CooperSurgical, Måløv, Denmark), according to the manufacturer’s manual. After the isolation process, the cells were centrifuged, and the top liquid layer was removed, leaving only the bottom 100-µL pellet. Finally, 15 µL of sperm-cell solution was added to 2 mL of Quinn’s Sperm Washing Medium (ref-art 1006 from Sage).

### 2.2. Disposable Microfluidic Device Architecture and the System Function

**Figure 1:**
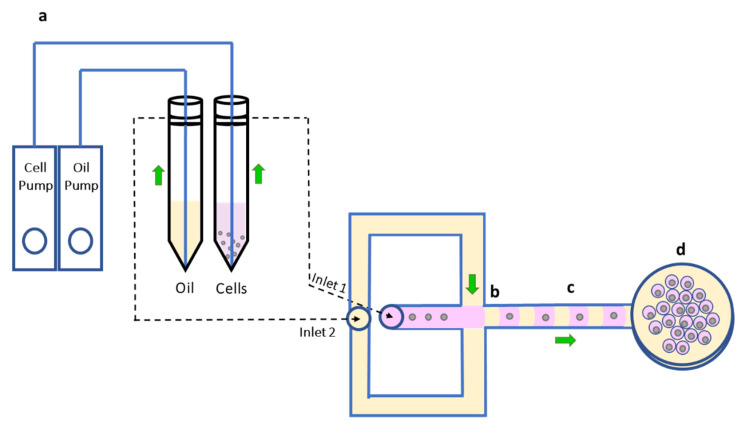
The microfluidic system for droplets formation, trapping each sperm cell in a separate microdroplet. (**a**) The pumps connect to the tubes; one tube contained oil (dSURF) and the other contained the cells in growth medium. (**b**) The T junction, where the droplets were formed by oil crossing. (**c**) The cells were trapped individually in droplets separated by oil and flowed outside from the channel. (**d**) The imaging petri dish was located at the end of the channel, where the droplets were collected. The oil ensured that the droplets did not merge with each other, creating a single-cell microenvironment for analysis and imaging.

The proposed microfluidic device for droplet formation is shown in Figure 1. The selected microfluidic chip (Fluidic 440, Microfluidic ChipShop, Jena, Germany) had eight channels with widths and heights of 50–80 μm both. In our experiments, we selected the 60 μm channel. As shown in Figure 1, each channel had two inlets and one outlet.

Two pumps (LineUp Series, Fluigent, Bicêtre, France) were connected to the conical centrifuge tubes with a total volume of 15 mL and to a pressure-pump source (FLPG Plus, Fluigent, Bicêtre, France). The first liquid contained the sperm cells in growth medium, which flowed into the channel from inlet 1. The second liquid was dSurf, a high-performance surfactant that maintained the liquid droplet stability, which flowed from inlet 2.

Concentration of sperm cells was 650,000 cells/mL. The place where the droplets were formed was the T junction, as indicated in Figure 1b. The sperm cells flowed into the T junction from the straight channel (marked in pink) and dSurf flowed into the T junction from both sides (marked in yellow), so that it ruptured the flow of the cell liquid from inlet 1. Therefore, the separated microdroplets were formed while in each of them, a single cell is trapped and thus separated from the other cells. At the end of the channel, there was only one outlet through which the microdroplets flowed to a petri dish where a rug of separated microdroplets was formed by self-assembly. After the droplet-rug formation was complete, mineral oil was poured on top to fill the petri dish, in order to prevent dehydration of the droplets, and keep the cells alive for a long time, as demonstrated.

Before droplet formation, the microchannel was filled with dSurf to remove the air inside the channel. To avoid backflow and prevent the dSurf from entering the cell pumps, a valve was placed at each of the tubes that connected the pumps and the channel. After dSurf filled the channel, the cell valve was opened, and the sperm cell liquid started flowing into the channel, where the microdroplets were formed. The average flow rate of the sperm cells was 2.5 µL/h, and the average flow rate of the dSurf was 5 µL/h.

### 2.3. Quantitative Phase Imaging

**Figure 2:**
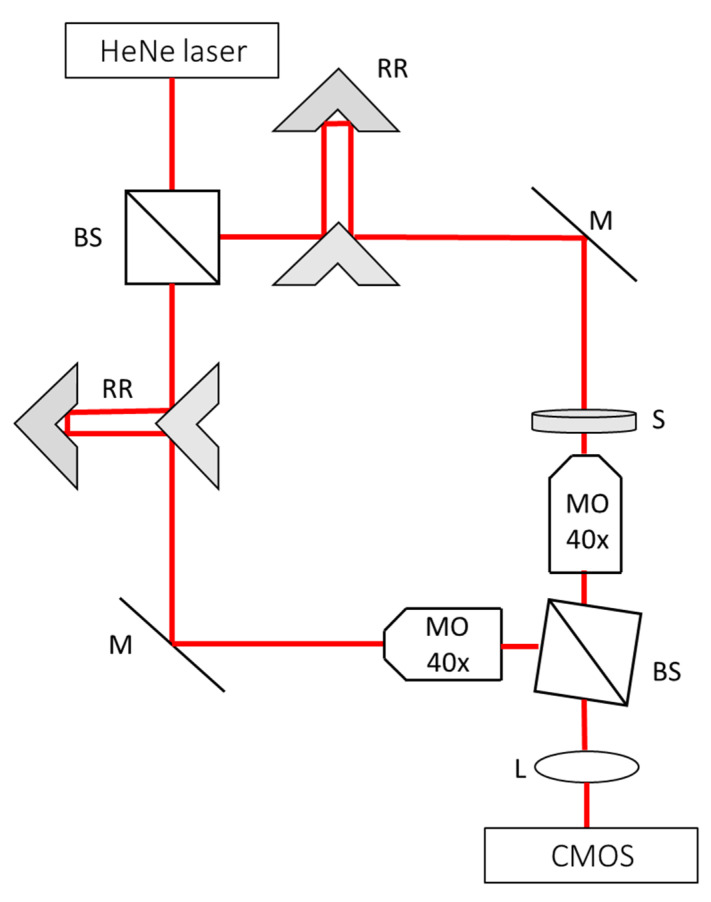
The quantitative phase microscopy arrangement. An off-axis Mach–Zehnder imaging interferometer. Beam-splitter (BS). Mirror (M). Two-mirror retroreflector (RRs). Sample (S) containing the cells trapped in microdroplets. Microscope objective (MO). Lens (L). Monochrome digital camera (CMOS).

For stain-free quantitative imaging of the microdroplet rug, we used the arrangements shown in Figure 2. The optical system was an off-axis Mach-Zehnder interferometer containing a Helium-Neon (HeNe) laser (wavelength 633 nm) that was split by the first beam splitter (BS) to a sample beam and a reference beam. The sample beam illuminated the rug of microdroplets and was magnified by a 40× microscope objective (Leica 440, 0.66 NA) and tube lens L (f = 200 mm) onto a CMOS camera (Thorlabs, DCx1545, Newton, NJ, USA). Similarly, the reference beam passed through a 40× microscope objective and combined with the sample beam by the second BS. Retroreflectors (RRs) enabled an optical-path match between the sample beam and the reference beam. The off-axis interferogram was recorded by the camera in a single exposure, and was then processed in the computer to the quantitative phase map of each cell. This process included a Fourier transform, cropping one of the cross-correlation terms, an inverse Fourier transform, and a two-dimensional-phase unwrapping on the phase argument of the resulting complex wave front [27].

Using the quantitative phase image, the various characteristics associated with each cell morphology could be assessed. First, the head shape should be oval and with a normal ratio of head width to length. Second, the acrosomal area should comprise 40–70% of the head area and contain no large vacuoles and no more than two small vacuoles, which should not occupy more than 20% of the head. Third, the midpiece should be approximately the length of the head, and its major axis should be a continuation of the head major axis. Finally, residual cytoplasm is considered an anomaly only when it exceeds one-third of the head [8,12,14].

### 2.4. Motility Analysis

To calculate the cell velocities, we tracked the cells for 10 min each for 5.5 h, taking a video of 10 frames with 50 ms time difference between each frame. We used these frames to calculate the cell velocity by dividing cell position difference and the time difference. Each time, we considered approximately 5 cells in the field of view.

### 2.5. Extracting the Sperm Cells from the Microdroplets

The removal of the sperm cells from the droplets was performed using a single-cell micromanipulator (MN-4 hydraulic manipulator, Narishigi, Japan) connected to a micropipette. After the OPD of the cell was obtained using the optical system, the clinician entered the chosen droplet with the micropipette and removed the sperm cell. Then, the chosen sperm cell was moved to a clean-medium droplet for further analysis, if needed, which could then be followed by an injection into an oocyte.

## 3. Results

### 3.1. Trapping Sperm Cells inside Separated Microdroplets

**Figure 3:**
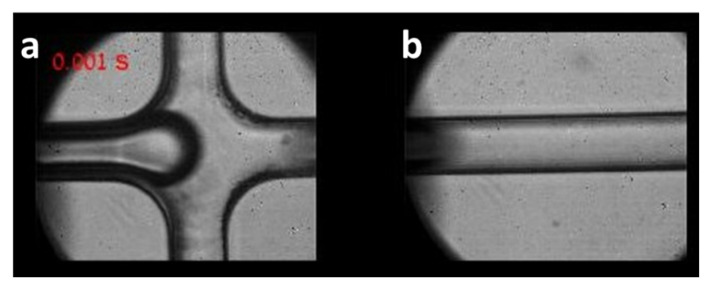
Microdroplet formation and flowing, as imaged by bright-field microscopy under 20× magnification. (**a**) At the T junction. (**b**) Further down the channel. See dynamics in Video S1.

Figure 3 and Video S1 present the microdroplet formation in the microchannel. The video was recoded with a fast camera (FASTCAM Mini AX200, Photron; square pixels of 20 μm each, 1024 × 1024 pixels) at 1000 frames per second. Figure 3a shows the microdroplet formation at the T junction, where the dSurf meets the cell liquid, and Figure 3b shows the microdroplets flowing down the channel. The size of the droplets and the distance between each droplet depended on the pressures and the velocities of these two stages, which were set manually by the pumps connected to the microchannel.

We first checked the initial concentration of the cells in the tubes. On one hand, we wanted to avoid many cells trapped in one microdroplet in order to allow separate analysis of each cell. On the other hand, lowering the cell concentration too much could result in empty droplets. We started with a concentration of 5 μL of sperm cells in 2 mL of medium, which resulted in many empty droplets and led to few sperm cells in the field of view. Then, the concentration of the sperm cells was slowly increased, and the best one was determined to be 12–15 μL of sperm cells in 2 mL of medium. Although in these concentrations some microdroplets have more than one cell, there are very few empty droplets, allowing the analysis of many cells in the field of view.

**Figure 4:**
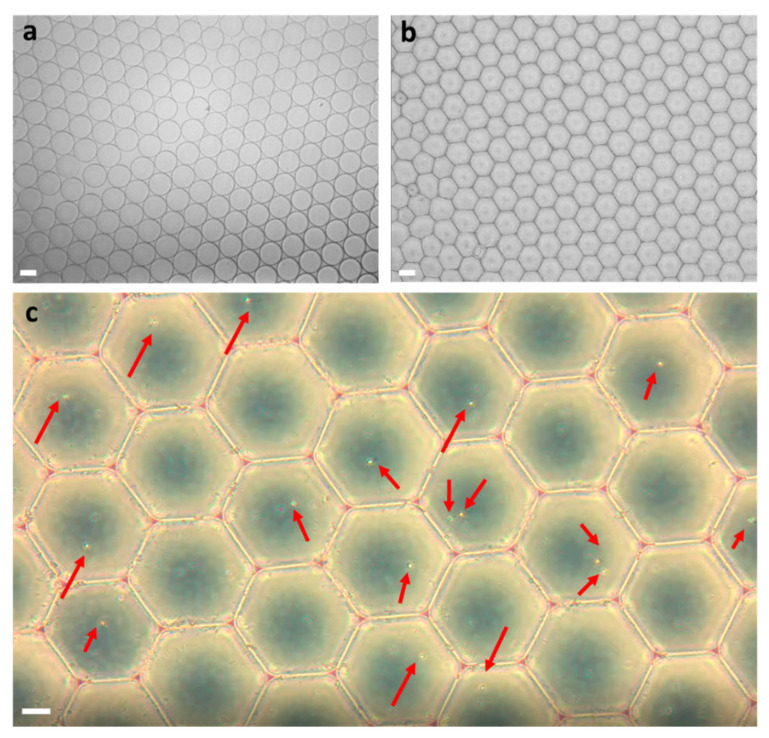
Microdroplets in the petri dish under bright-field microscopy. (**a**) The droplets as they exited the microfluidic channel to the petri dish. (**b**) The droplets after they stabilized to form hexagon shapes. (**c**) The sperm cells inside the droplets are marked by the red arrows. Most droplets contained a single sperm cell, and several contained either two sperm cells or none. White scale bars represent 40 µm in (**a**,**b**) and 20 µm in (**c**).

Figure 4a,b show the shape of the microdroplets after they leave the microchannel. When the droplets exited the microfluidic channel, their shape was a perfect sphere (Figure 4a), but after a few seconds, the droplets flowed closer to each other, stabilized, and formed a hexagonal shape (Figure 4b). Figure 4c shows the sperm cells inside the droplets at the end of the droplet formation process. As can be seen, due to the tradeoff between the number of cells inside each droplet and the number of empty droplets, there were some empty droplets and a few droplets with two cells, but most of the droplets contained only a single cell.

### 3.2. Motility Analysis of the Cells

**Figure 5:**
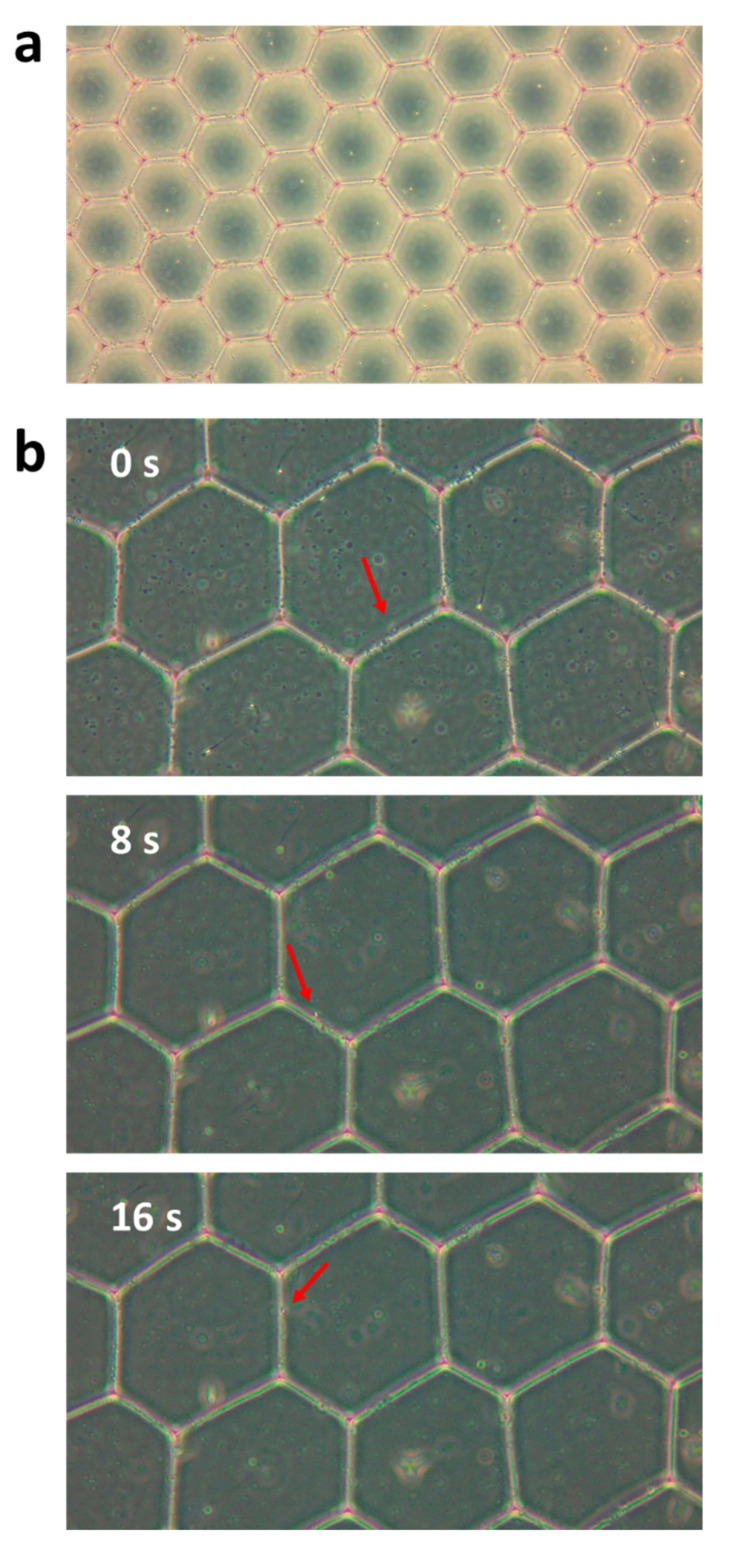
Dynamic bright-field imaging of the cells trapped in the microdroplets. (**a**) Under 20× magnification. See dynamics in Video S2. (**b**) Under 40× magnification. See dynamics in Video S3. White scale bars represent 40 µm in (**a**) and 20 µm in (**b**).

**Figure 6:**
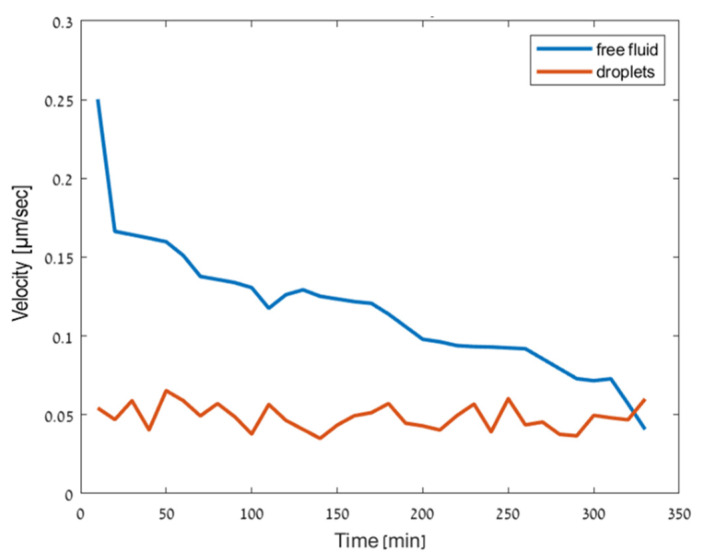
The velocity of the cells over 5.5 h, inside the microdroplets and in free medium.

Dynamically swimming sperm cells inside the droplets are presented in Figure 5 and Videos S2 and S3. We inspected the velocity of the cells over 5.5 h, as tracked each 10 min. The result is presented by the red curve in Figure 6. Motile sperm cells with head and tail rotation were seen even after 5.5 h due to the seal created by the oil. Inside the droplets, the sperm cells swam in a different way than their usual progressive movement in free medium. Inside the droplets, the cell heads tended to be in contact with the edges of the droplets [28]. For this reason, the velocities of the sperm cells inside the droplets were lower than the velocities in free medium. It can be seen from Figure 6 that after 5.5 h, the cells swimming in free medium had slowed down, with velocities in the range of 0.05–0.25 µm/s, while the cells that were trapped in the droplets maintained their velocity around 0.05 µm/s throughout the entire experiment. The ratio of the number of motile cells and the total number of cells in the free medium at *t* = 0 h was 0.2, while at *t* = 5.5 h, it was 0.077. The ratio of the motile cells from the total cells in the microdroplets at *t* = 0 h was 0.2 while at *t* = 5.5 h, it was 0.074. These results show that even though the free medium contained more cells in the field of view, the ratio between the number of moving cells and the total number of cells in the field of view stayed approximately the same in both the free medium and the microdroplets, implying that the droplet compartmentalization did not affect the viability of the cells.

This feature of microdroplet trapping yields an ideal microenvironment for morphological measurement of the cells, i.e., without cells occluding each other during morphological imaging, and also significantly eases the trapping of the cells via micropipette after their morphological assessment.

### 3.3. Noise Levels in the Quantitative Phase Imaging System

**Figure 7:**
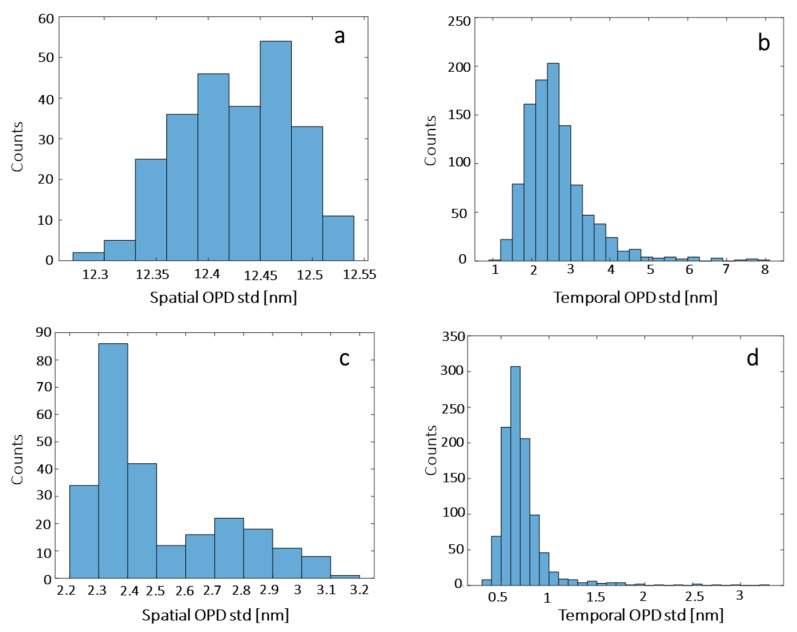
OPD noise-level distribution for: (**a**,**b**) a microdroplet sample, and for (**c**,**d**) an empty sample. (**a**,**c**) Spatial noise levels. (**b**,**d**) Temporal noise levels.

The OPD sensitivities across an image and between the images are known as the system spatial and temporal noise levels, respectively [27]. We recorded two different samples, a plate with no droplets and a plate with a rug of droplets and mineral oil above it. In each scenario, we recorded 250 interferograms for 10 s, and then processed them to obtain their OPD maps. Next, we calculated the temporal noise level as the standard deviation (std) per single diffraction-limited spot across the 250 OPD maps, and the spatial noise level as the std across the OPD map. Figure 7a,b present the distributions of these std values for the microdroplet sample, with an average spatial std of 12.428 nm and an average temporal std of 2.64 nm, while Figure 7c,d present the distributions of these std values for the empty sample, with an average spatial std of 2.51 nm and an average temporal std of 0.72 nm, demonstrating 4.95× increase of the spatial noise levels and 3.66× increase of the temporal noise levels when imaging through the droplets.

### 3.4. Morphological Analysis of the Cells by Quantitative Phase Imaging

**Figure 8:**
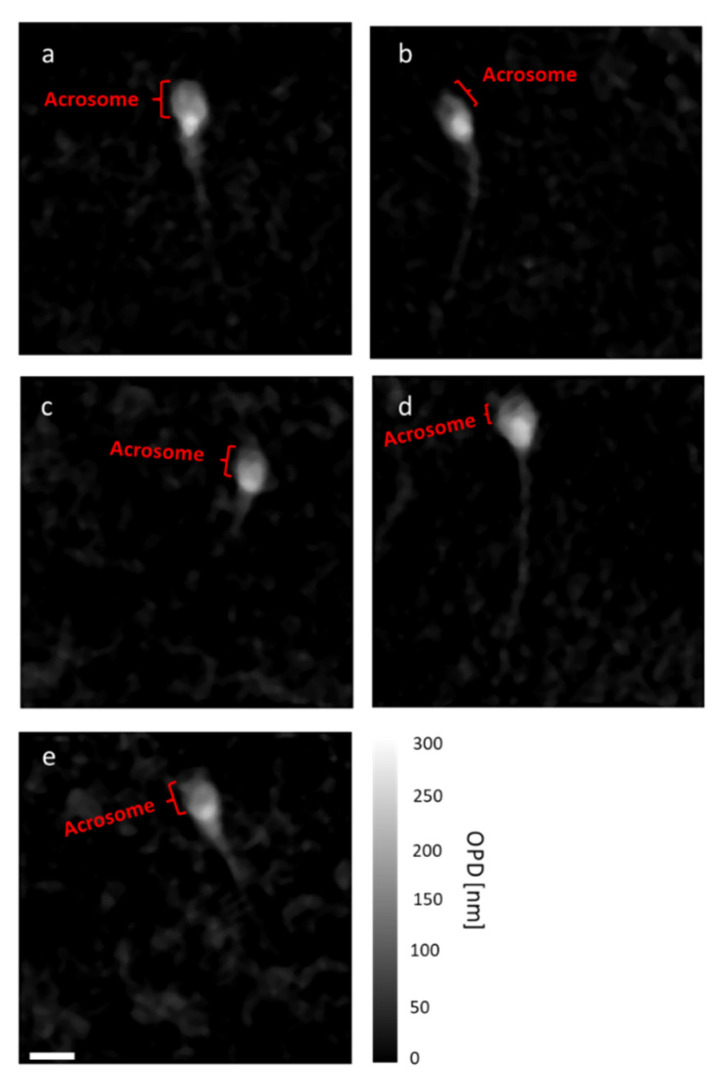
(**a**–**e**) OPD maps of cells a-e in Table 1, captured while dynamically swimming inside the microdroplets. The white scale bar represents 5 µm. Color bar represents OPD values in nm.

We used quantitative phase microscopy for measuring the OPD maps of the cells inside the microdroplets. Since the rug of the droplets was covered by mineral oil, the droplets flattened, and there was no visible effect of the droplet curvature in the OPD map. As shown in Figure 8, the OPD maps showed the head of the sperm cells clearly with the acrosomal area. Based on various morphological parameters obtained from the OPD maps, a determination was made whether a sperm cell was suitable for fertilization [12,13]. Table 1 presents five morphological parameters obtained from the cells in Figure 8. A potent sperm cell should have acrosomal region comprising 40–70% of the head area. This value can only be measured when the cell is chemically stained, which is not allowed during human IVF, or when using stain-free quantitative phase microscopy, as demonstrated by the acrosome-head ratios in Table 1. Another parameter is the radii ratio. The accepted normal ratio of the head width to length is 3:5 [8,12]. According to Table 1, only cells a, b, and c should be selected, since they meet these morphological requirements. These three cells had acrosomal–head ratios of 40–70%, while the other cells had acrosomal–head ratios of less than 40%.

### 3.5. Extraction of Sperm Cells from the Microdroplets

**Figure 9:**
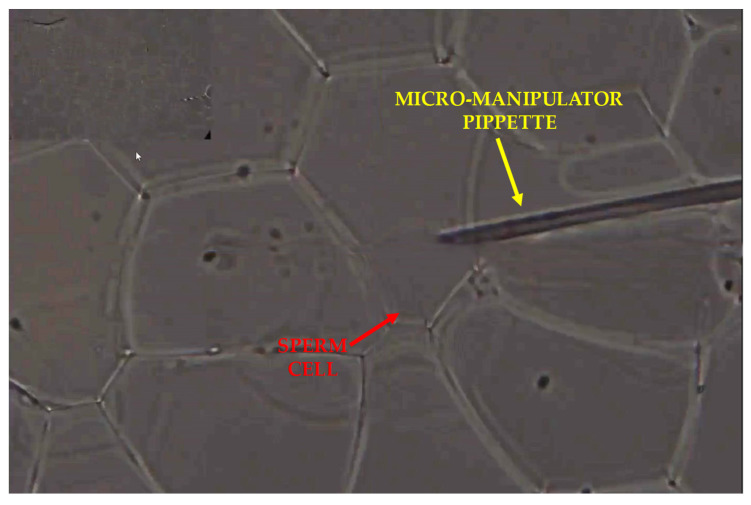
Extraction of the sperm cells from the droplet using a micropipette and their insertion into an empty droplet, collecting all potent sperm cells for later oocyte injection, after performing the quantitative phase microscopy morphological analysis. See dynamics in Video S4.

After we decided which sperm cell was the most suitable one for fertilization, we removed the cell from the microdroplet, and either injected it directly into the oocyte or inserted it into an empty droplet for later injection. Figure 9 and Video S4 present the cell extraction and insertion process using a cell micromanipulator. As shown in Video S4, first the clinician focused on the chosen cell and then lowered the micropipette to enter the microdroplet. The pipette had to gently penetrate through two layers: the mineral oil layer and then the droplets themselves. Inside the selected droplet, the cell was slowly sucked into the pipette without destroying the surrounding droplets. Afterwards, the clinician released the cell into an empty droplet. This droplet could contain either all selected potent cells or only one cell. At a later time, these selected cells could be used for oocyte injection.

## 4. Discussion

The rate of the droplet formation needs to be fast in order to capture only one sperm cell in each microdroplet. If the flow is too slow, several cells could enter the droplet, which complicates any later analysis. In the preliminary experiments without the cells (i.e., using only water and dSurf), the ideal velocities and pressures for droplet formation (shown in Video S1) were found. We optimized the flow rate to be 40–50 droplets/second. At this rate, the droplets were the same size, resulting in a stable flow. The space between the droplets was created by using dSurf in the channel instead of regular mineral oil. When the droplets exited the channel, they had a spherical shape, as can be seen in Figure 4a, but shortly after, they approached each other in the petri dish to form a rug structure, and then each droplet formed a hexagonal shape due to self-assembly, still without mixing with each other, as can be seen in Figure 4b. This was the stable form of the droplets. In this form, the rug of the droplets was subjected to various forces and could move around the petri dish, which was an undesirable condition. We therefore used mineral oil poured on top of the droplet rug, which flattened and fixed the droplets in place, as shown in Figure 4c. This step was also beneficial for quantitative phase imaging, due to flattening the background phase.

As shown in Videos S2 and S3, inside the droplets, the cells tended to swim near the edges of the droplets but failed to break the droplet. In both videos, the immotile cells were usually at the center of the droplets, and the tails of these cells could hardly be seen. The cell motility overtime was quantified and compared to the swimming of sperm cells in a free medium, demonstrating that the cells trapped in the microdroplets were motile for a longer period (more than 5.5 h).

The temporal and spatial noise levels of quantitative phase microscopy through the droplets were measured to determine the smallest OPD change that could be detected, and both were in the nanometric range with average values of 12.4 nm and 2.5 nm for the spatial and temporal noise levels, respectively. Quantitative phase imaging was then used for the cell morphological assessment inside the microdroplets. We have demonstrated that we could characterize the head shape and the acrosome area through the OPD image of the cell, and could differentiate between the normal and pathological cells.

## 5. Conclusions

We have introduced a new method for quantitatively analyzing and selecting sperm cells for fertilization, which is both rapid and simple. It gives the embryologist the ability to directly analyze the morphology and motility of the sperm cells over time, while cells remain in the imaging field of view, before trapping them with a micropipette for oocyte injection.

Individual sperm cells were trapped in microdroplets during a process that combined two liquids, the cell medium and dSurf. Depending upon the initial concentration of cells, typically a single cell could be trapped in each microdroplet without the ability to escape after analysis and before selection. Due to the close microenvironment, the cells were kept motile inside the droplet for more than 5.5 h, resulting in a long time frame, which is useful for various assays. The quantitative phase maps of the cells, as acquired inside the droplets, assisted in differentiating between normal and pathological cells via stain-free quantitative-phase morphological analysis. We also demonstrated the extraction of single sperm cells from the microdroplets without damaging the surrounding droplets using a cell micromanipulator that has typically been used in clinical ICSI procedures. Hence, we demonstrated that analyzing and selecting individual sperm cells within microdroplets enhanced the selection process of cells for fertilization via stain-free quantitative phase microscopy and morphological analysis. The proposed method can be integrated into practical ICSI procedures, making sperm analysis in ICSI more quantitative and less subjective and, therefore, potentially increasing this procedure success rates.

## 6. Patents

A provisional patent application has been submitted.

## Figures and Tables

**Table 1:** Morphological parameters extracted from the OPD maps of the cells.

	Acrosome–Head Ratio [%]	Radii Ratio	Midpiece–Head Ratio	Vacuole Presence	Residual Cytoplasm Presence
Cell a	59.64	0.57	1.24	No	No
Cell b	47.3	0.56	1.11	No	No
Cell c	65.04	0.63	1.13	No	No
Cell d	31.01	0.7	1.16	No	No
Cell e	38.96	0.48	1.32	No	No

## Data Availability

All data can become available for non-commercial goals upon a reasonable request.

## Supplementary Materials

The following are available online at https://www.mdpi.com/article/10.3390/cells10123317/s1. Video S1: Microdroplet formation and flowing in the channel. Video S2: Dynamic bright-filed imaging of the cells trapped in the microdroplets under 20× magnification. Video S3: Dynamic bright-filed imaging of the cells trapped in the microdroplets under 40× magnification. Video S4: Extraction of the sperm cells from the droplets using a micropipette and cell insertion into an empty droplet.
